# A frameshift insertion in *FA2H* causes a recessively inherited form of ichthyosis congenita in Chianina cattle

**DOI:** 10.1007/s00438-021-01824-8

**Published:** 2021-10-02

**Authors:** Joana G. P. Jacinto, Irene M. Häfliger, Inês M. B. Veiga, Anna Letko, Arcangelo Gentile, Cord Drögemüller

**Affiliations:** 1 Department of Veterinary Medical Sciences, University of Bologna, Ozzano Emilia, 40064 Bologna, Italy; 2 Institute of Genetics, Vetsuisse Faculty, University of Bern, 3001 Bern, Switzerland; 3 Institute of Animal Pathology, Vetsuisse Faculty, University of Bern, 3012 Bern, Switzerland

**Keywords:** Bovine, Genodermatoses, Fatty acid 2-hydroxylase, Precision medicine, Skin, Urolithiasis

## Abstract

The aim of this study was to characterize the phenotype and to identify the genetic etiology of a syndromic form of ichthyosis congenita (IC) observed in Italian Chianina cattle and to estimate the prevalence of the deleterious allele in the population. Sporadic occurrence of different forms of ichthyosis including IC have been previously reported in cattle. However, so far, no causative genetic variant has been found for bovine IC. Nine affected cattle presenting congenital xerosis, hyperkeratosis and scaling of the skin as well as urolithiasis and cystitis associated with retarded growth were examined. Skin histopathology revealed a severe, diffuse orthokeratotic hyperkeratosis with mild to moderate epidermal hyperplasia. The pedigree records indicated a monogenic recessive trait. Homozygosity mapping and whole-genome sequencing allowed the identification of a homozygous frameshift 1 bp insertion in the *FA2H* gene (c.9dupC; p.Ala4ArgfsTer142) located in a 1.92 Mb shared identical-by-descent region on chromosome 18 present in all cases, while the parents were heterozygous as expected for obligate carriers. These findings enable the selection against this sub-lethal allele showing an estimated frequency of ~ 7.5% in Chianina top sires. A sporadic incidence of mild clinical signs in the skin of heterozygous carriers was observed. So far, pathogenic variants affecting the encoded fatty acid 2-hydroxylase catalyzing the synthesis of 2-hydroxysphingolipids have been associated with myelin disorders. In conclusion, this study represents the first report of an *FA2H*-related autosomal recessive inherited skin disorder in a mammalian species and adds *FA2H* to the list of candidate genes for ichthyosis in humans and animals. Furthermore, this study provides a DNA-based diagnostic test that enables selection against the identified pathogenic variant in the Chianina cattle population. However, functional studies are needed to better understand the expression of FA2H in IC-affected Chianina cattle.

**Supplementary Information:**

The online version contains supplementary material available at 10.1007/s00438-021-01824-8.

## Introduction

The aim of this study was to report a series of nine cases of IC in Chianina cattle, to characterize the clinicopathological phenotype and finally to present the results of the genetic analysis that evidenced a homozygous frameshift variant in bovine *FA2H* gene. Moreover, the prevalence of the deleterious allele in a selected population of Chianina sires is also estimated.

Genodermatoses are sporadic inherited disorders of the skin that both in humans and in livestock animals mostly follow a monogenic mode of inheritance (Leeb et al. 2017; Jacinto et al. 2020; Pope 2020).

In human medicine, the concept of ‘genodermatosis with skin fragility’ was recently introduced by adding other genetic disorders with skin fragility such erosive or hyperkeratotic disorders (Pope 2020). Inherited ichthyosis characterized by an abnormal terminal keratinocyte differentiation belongs to this group of skin fragility disorders encompassing a clinically, pathologically and heritably heterogeneous presentation with a thickened stratum corneum resulting in localized or generalized scaling (Marukian and Choate 2016).

In human medicine, the classification of the different forms of ichthyosis is based on clinicopathological manifestations and mode of inheritance, being divided in two main types: non-syndromic forms when clinical findings are limited to the skin, and syndromic forms in case additional organs are involved (Oji et al. 2010). In this respect, ichthyosis has been associated to pathogenic variants in more than 30 genes that are involved in several cellular functions, such as DNA repair, lipid biosynthesis, adhesion and desquamation (Oji et al. 2010). In particular, recent advances have reinforced the causative role of mutations in genes encoding proteins essential to the formation of the hydrophobic barrier (Marukian and Choate 2016).

In domestic animals, ichthyosis has been described in dogs (Credille et al. 2009; Grall et al. 2012; Metzger et al. 2015; Bauer et al. 2017; Casal et al. 2017), pigs (Wang et al. 2019), sheep (Câmara et al. 2017) and cattle (Charlier et al. 2008; Woolley et al. 2019; Eager et al. 2020). Furthermore, it has also been reported in greater kudu calves (Chittick et al. 2002). While in sheep, pigs and greater kudu the underlying genetic cause of this condition has not been determined, in dogs pathogenic variants have been identified in five different candidate genes associated with the phenotype, four breed specific recessive inherited forms (*TGM1*, *SLC27A4*, *PNPLA1*, *NIPAL4*) (OMIA 000546-9615; OMIA 001973-9615; OMIA 001588-9615; OMIA 001980-9615) as well as a single dominant inherited form in a single affected dog (*ASPRV1*) (OMIA 002099-9615).

In Chianina, Shorthorn and Polled Hereford cattle, a form of ichthyosis named ichthyosis fetalis, which resembles the Harlequin-type ichthyosis described in human medicine, has been associated with recessively inherited mutations in *ABCA12* (OMIA 002238-9913) (Charlier et al. 2008; Woolley et al. 2019; Eager et al. 2020). Affected calves are stillborn or die within the first days after birth and the skin is diffusely covered with large horny plates separated by deep fissures and resembling a ‘leather cuirass’. Furthermore, eversion at mucocutaneous junctions provokes eclabium and ectropion (Chittick et al. 2002; Molteni et al. 2006).

In Chianina cattle, a second less severe form of ichthyosis, named ichthyosis congenita (IC), has also been described (Testoni et al. 2006) and in subsequent time repeatedly presented to the authors. Animals with IC show milder but comparable lesions to those of ichthyosis fetalis. It is clinically characterized by a more or less extended scale-like hyperkeratosis and multifocal alopecic areas, and histopathologically by a diffuse lamellar orthokeratotic hyperkeratosis. The underlying genetic cause of this form of syndromic form of ichthyosis associated with retarded growth is unknown.

## Methods

### Animals

This study did not require official or institutional ethical approval as it was not experimental, but rather part of clinical and pathological veterinary diagnostics. All animals in this study were examined with the consent of their owners and handled according to good ethical standards. It deals with a total of 129 Chianina cattle, including 9 IC-affected animals, 4 dams, 6 sires and 110 artificial insemination (AI) top sires. The tenth affected animal included in the study (case 10) was the one previously reported by Testoni et al. (2006), whose blood had at that time been frozen and therefore had remained available for genetic studies.

### Clinical and pathological investigations

Eight calves (cases 1–8) and one heifer (case 9) presenting cutaneous hyperkeratosis and retarded growth were recorded by the teaching hospital of the Department of Veterinary Medical Sciences, University of Bologna between 2005 and 2020 (Online Resource 1). The mean age of record of the calves was 2.6 months (minimum–maximum: 2 days–7 months), whereas the heifer was 18 months. The mean age at death was 11.2 months (natural death, euthanasia or slaughtering). All affected animals and one dam (case 8’s dam) were thoroughly clinically examined. Information related to the skin condition of the other dams as well as of the sires were obtained by interviewing the owners or the breeders’ association, respectively.

A parasitological test for detection of ectoparasites and fungi infection was performed on case 8’s dam.

Skin biopsies using an 8 mm punch were obtained from seven affected animals (cases 1–3 and cases 6, 8, 9) and from case 8’s dam. The collected samples were fixed in 10% neutral buffered formalin, trimmed, processed, embedded in paraffin wax, sectioned at 4 µm, and stained with hematoxylin and eosin (H&E) for further histological evaluation. Two affected animals (cases 2 and 8) were submitted to necropsy.

### Pedigree design

Pedigree analysis was performed using Pedigraph version 2.4 software (Department of Animal Science, University of Minnesota, USA).

### DNA extractions

Genomic DNA was extracted from the IC-affected animals (EDTA blood samples), four related dams (EDTA blood samples) and six related sires (semen) using Promega Maxwell RSC DNA system (Promega, Dübendorf, Switzerland). Furthermore, genomic DNA was also obtained from semen of 110 Chianina AI top sires with the same methodology.

### SNP array genotyping and homozygosity mapping

High‐density SNP genotyping was carried out for seven cases (cases 1–7) and eight obligate carriers (three dams and five sires) (Online Resource 1) on the Illumina BovineHD BeadChip array including 777.962 SNPs. All given SNP positions correspond to the bovine ARS-UCD1.2 genome assembly. The PLINK v1.9 software (Chang et al. 2015) was used to perform basic quality filtering of the dataset. Even though no sample was excluded, a total of 146.440 variants were removed owing to minor allele thresholds. The total genotyping rate was approximately 0.98. With a total of 631.522 remaining markers, homozygosity mapping was performed for the 7 IC-affected animals using the software PLINK v1.9 (Purcell et al. 2007) with the commands *‐‐homozyg‐kb* 100 (considering homozygous segments of at least 100 kb), *‐‐homozyg‐match* 0.95 (for allelic matching between both cases) and *‐‐homozyg‐group* (for generating an overlap‐file), resulting in shared runs of homozygosity (ROH) indicating chromosomal region of identity-by-descent (IBD).

### Whole-genome sequencing and variant calling

WGS using the Illumina NovaSeq6000 (Illumina Inc., San Diego, CA, USA) was performed on the genomic DNA of two affected calves (cases 1 and 6). The sequenced reads were mapped to the ARS‐UCD1.2 reference genome, resulting in an average read depth of approximately 18.2× in case 1 and 17.9× in case 6, and single-nucleotide variants (SNVs) and small indel variants were called (Rosen et al. 2020). The applied software and steps to process fastq files into binary alignment map (BAM) and genomic variant call format files were in accordance with the 1000 Bull Genomes Project processing guidelines of run 7 (Hayes and Daetwyler 2019), except for the trimming, which was performed using fastp (Chen et al. 2018). Further preparation of the genomic data was done according to Häfliger et al. 2020 (Häfliger et al. 2020). To find private variants, we compared the genotypes of the two calves with 597 cattle genomes of various breeds that had been sequenced in the course of other ongoing studies and that are publicly available (Online Resource 2) in the European Nucleotide Archive (SAMEA7690197 and SAMEA7690198 are the samples accession number of case 1 and case 6, respectively; http://www.ebi.ac.uk/en). The filtered list of remaining variants were further checked for their occurrence in a global control cohort of 4110 genomes of a variety of breeds (Hayes and Daetwyler 2019). Integrative Genomics Viewer (IGV) (Robinson et al. 2017) software was used for visual inspection of genome regions containing possible candidate genes.

### Variant validation and genotyping via Sanger sequencing

PCR and Sanger sequencing were used to confirm the WGS results and to perform targeted genotyping for the identified *FA2H* frameshift insertion variant (18:2205625C>CG). All IC-affected animals, four available dams and six sires, as well as 113 AI top sires that included three fathers of the studied cases, were genotyped for the identified variant. Also the case reported by Testoni in 2006 (case 10) (Testoni et al. 2006) was genotyped. Primers were designed using the Primer-BLAST tool (Ye et al. 2012). After amplification with AmpliTaqGold360Mastermix (Thermo Fisher Scientific) the purified PCR products were directly sequenced on an ABI3730 capillary sequencer (Thermo Fisher Scientific). The primer sequences used were the following: 5′-AAATTCCTGGTT-GGGGAGCC-3′ (forward primer) and 5′-CTCGACAACGAGACGCACC-3′ (reverse primer). The sequence data were analyzed using Sequencher 5.1 software (GeneCodes).

## Results

### Clinical phenotype

**Fig. 1:**
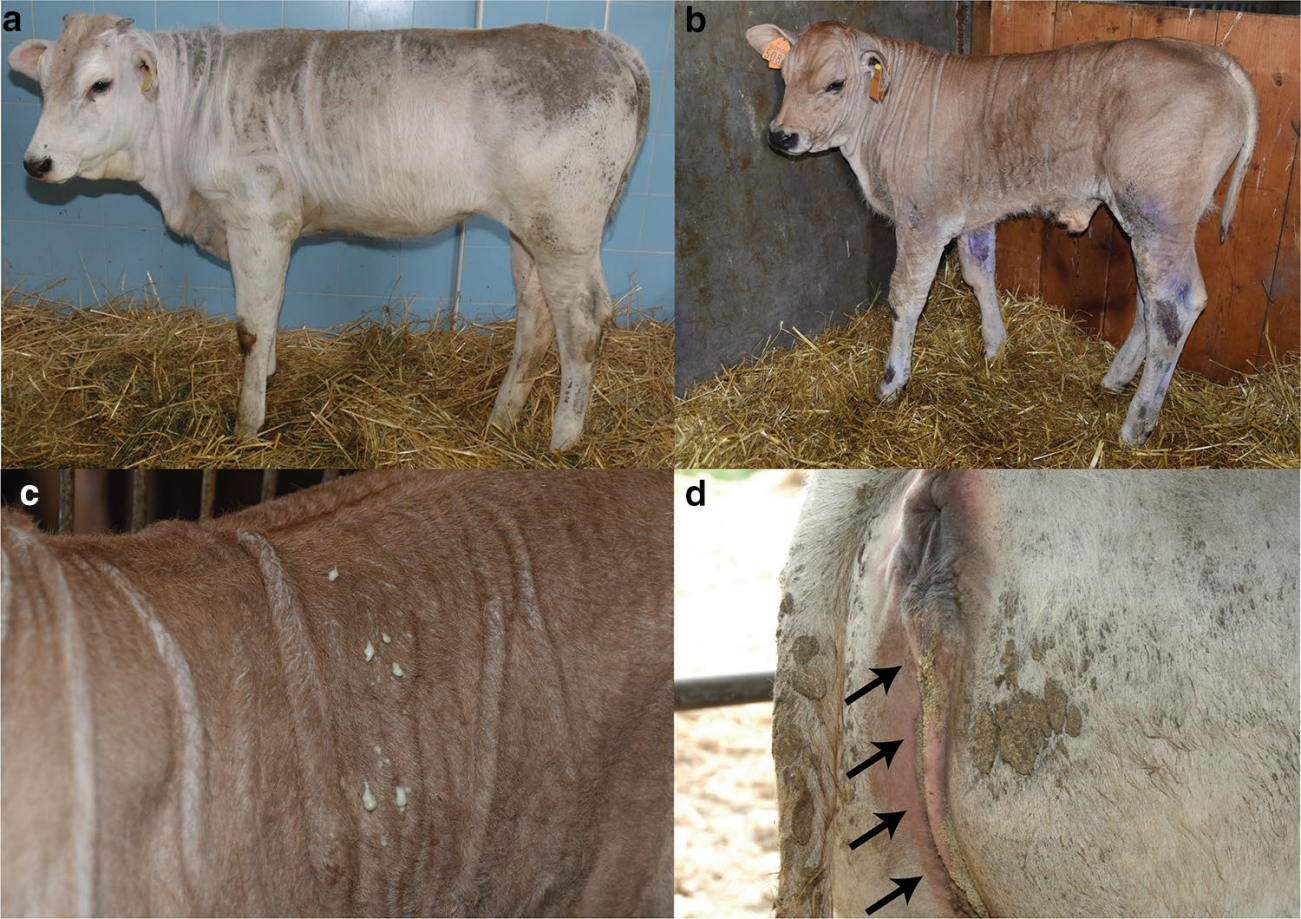
Clinical characterization of Chianina cattle affected by ichthyosis congenita. **a** Note the dry, greyish skin with scale-like hyperkeratosis over most of the body surface (case 1). **b** Note the multiple wrinkles, folds and wounds secondary to the hyperkeratosis (case 8). **c** Higher magnification of **b** from the skin of the thoracic region. Note the pyoderma. **d** Note the urolithiasis characterized by the presence of small stones and crystals (arrows) on the peri-genital region (case 7)

All patients (case 1–9) showed a more or less extended skin xerosis, hyperkeratosis and scaling besides a retarded growth. In the affected area the skin was dry and greyish with scale-like hyper-keratosis, and the most severe lesions were present at the level of trunk and neck (Fig. 1a). The coat was dull and bristly. Moreover, multifocal alopecic lesions were noticed, mostly affecting the muzzle, eyelids, ears, and inner region of limbs. The youngest calves displayed multiple wrinkles and folds (≤ 1 month of age) (Fig. 1b). No abnormalities were observed at the level of the mucocutaneous junctions. One of the animals (case 8) also showed secondary wounds and pyodermitis (Fig. 1c). Urolithiasis evidenced by the presence of small stones and crystals in the perigenital region (Fig. 1d) accompanied the cutaneous disease in cases 1, 7, 8 and 10. A hypoglycemic and hypothermic crisis that provoked the death of case 8 during the winter season was interpreted as a secondary phenomenon of imbalanced thermoregulation capacity. No abnormalities were registered at the level of the cardiovascular, respiratory, musculoskeletal, and nervous systems in any animals.

Interestingly, case 8's dam showed mild localized xerosis, hyperkeratosis and scaling in the region of the rump. Unfortunately, since in most cases the parents of the affected animals had already been slaughtered, we could not evaluate the phenotype in more of these obligate carriers. However, we did see a total of three other confirmed *FA2H* heterozygous Chianina cattle and they were clinically normal.

Based on the clinical observations, the affected animals were consequently suspected to suffer from IC as described in this breed in 2006 (Testoni et al. 2006). A similar diagnosis was advanced also for the dam of case 8, although in a very mild form. For this animal the differential diagnosis of ectoparasitosis and fungi infection were excluded on the base of a parasitological test.

### Pathological phenotype

**Fig. 2:**
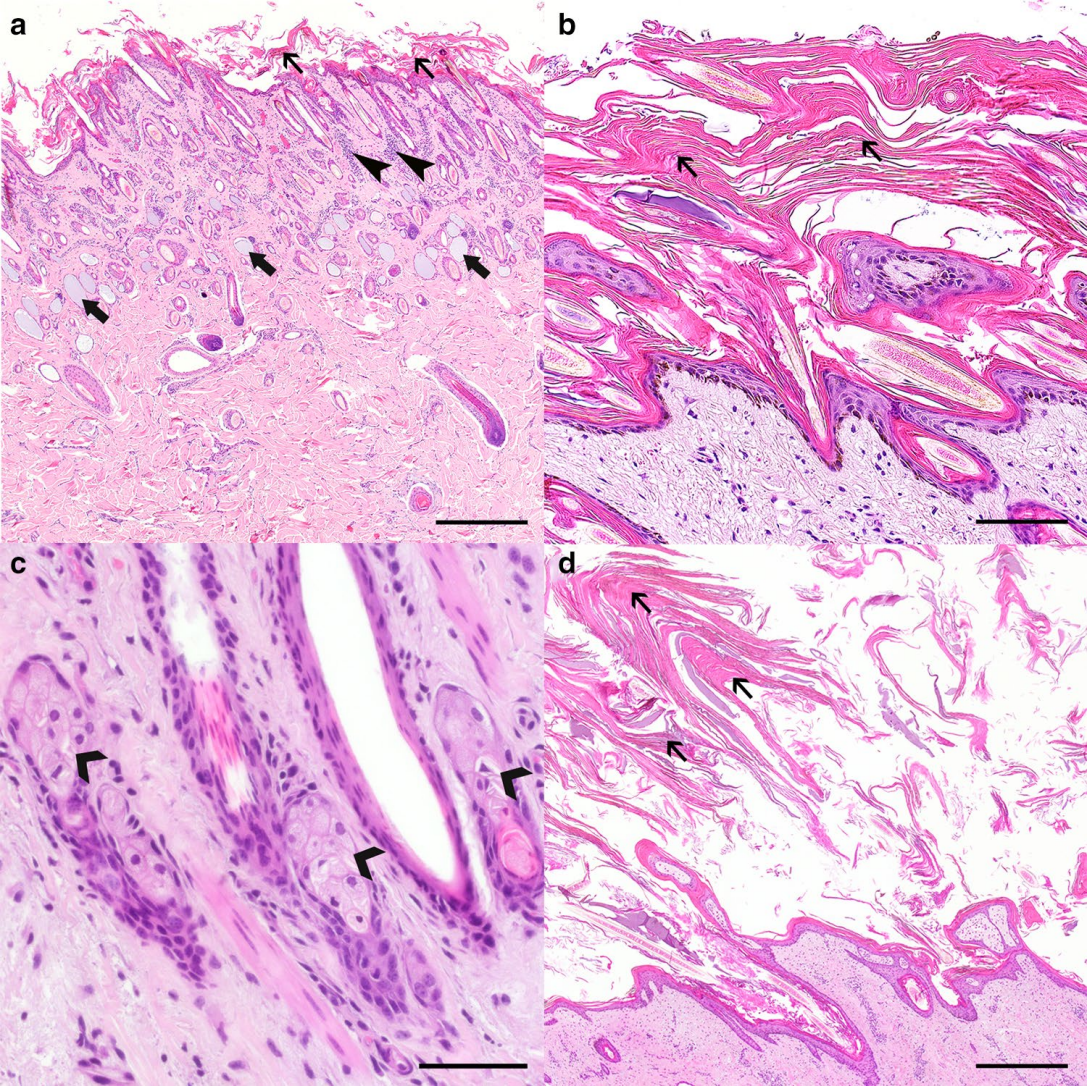
Histology of the skin lesions displayed by a IC-affected Chianina calf (**a**–**c**) and its dam (**d**). **a** The epidermis of the calf (case 8) is irregular and mildly hyperplastic, with a thick overlying stratum corneum composed of abundant orthokeratotic, lamellar keratin scales (thin arrows). The sebaceous glands are not noticeable at this magnification, and the sweat glands are often dilated and filled with basophilic, homogeneous material (large arrows). Occasional interstitial inflammatory infiltrates can be observed in the superficial dermis (thin arrowheads). H&E staining, 500 µm. **b** Detail of the abundant orthokeratotic, lamellar keratin scales (thin arrows) (case 8). H&E staining, 100 µm. **c** Detail of the sebaceous glands. Some sebocytes display intracytoplasmic, spindle-shaped, optically empty clefts (large arrowheads) (case 8). H&E staining, 50 µm. **d** A severe orthokeratotic hyperkeratosis (thin arrows) could also be observed in the dam from case 8. H&E staining, 500 µm

Histological analysis of the biopsies from the cutaneous lesions revealed a severe, diffuse orthokeratotic hyperkeratosis with mild to moderate epidermal hyperplasia (Fig. 2a, b). Serocellular crusts, serum lakes, and plant material were occasionally present among the abundant keratin scales. The superficial dermis displayed multifocal, moderate eosinophilic infiltrates, as well as a mostly perivascular, moderate infiltration with plasma cells and lymphocytes (Fig. 2a). Also, it was possible to observe the presence of intracytoplasmic, spindle-shaped, optically empty clefts within the sebocytes (Fig. 2c) in several of the affected animals, while the remaining adnexal structures were unremarkable. Similar findings were observed histologically in the punch biopsies taken from the dam of case 8 (Fig. 2d). These findings were consistent with the clinical diagnosis of IC.

Moreover, post-mortem examination of three cases (cases 1, 8 and 10) revealed inflammation of the urinary bladder (cystitis).

### Genetic analysis

**Fig. 3:**
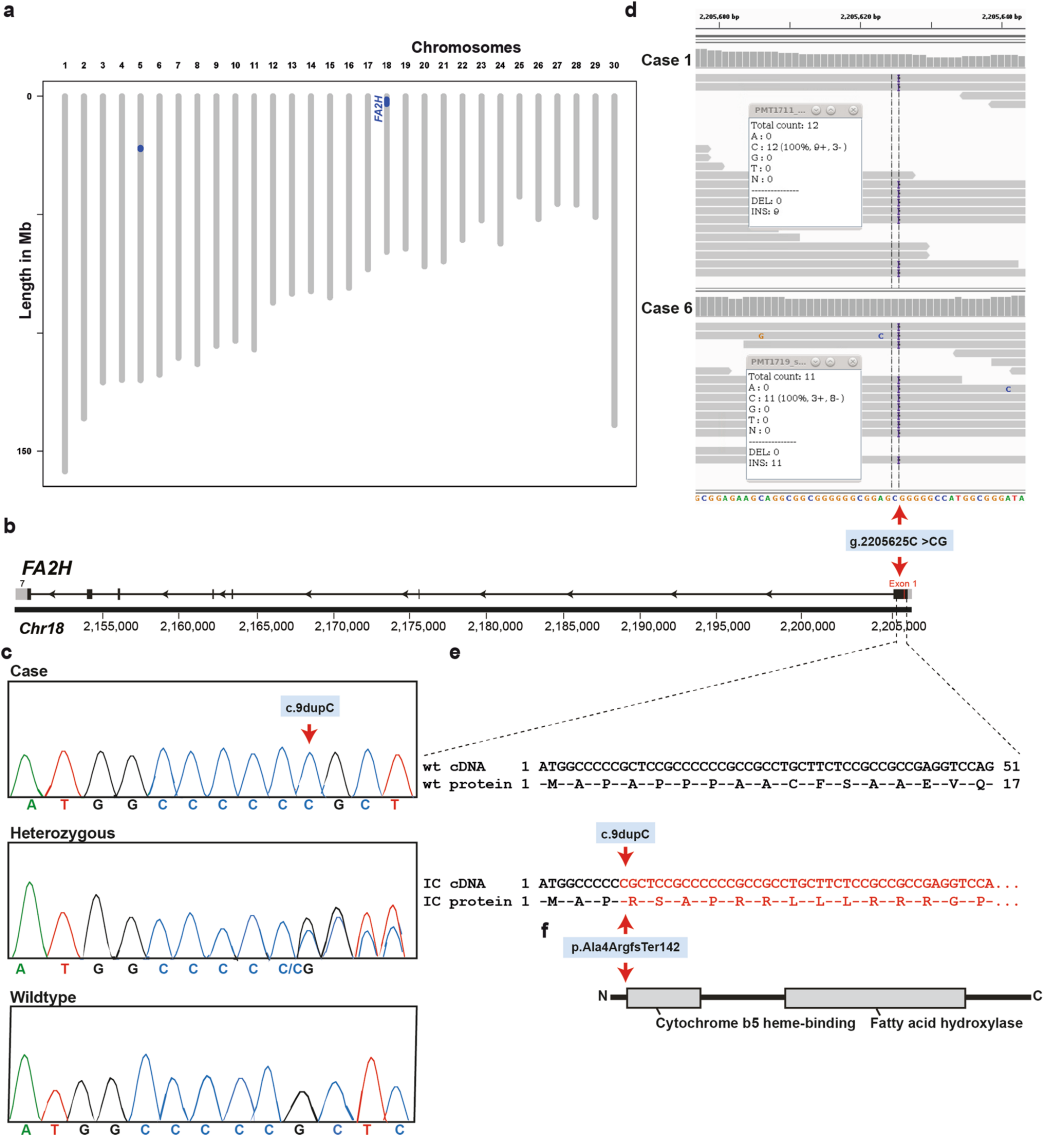
Ichthyosis congenita (IC) *FA2H* frameshift variant in Chianina cattle. **a** Genetic mapping of the IC locus in the cattle genome. The two regions of shared homozygosity of seven cases are displayed in blue. Note that the largest segment of 1.92 Mb on chromosome 18 containing the, *FA2H* gene. **b** *FA2H* gene structure showing the variant located in exon 1. **c** Electropherograms of a case, heterozygous and wild-type genotypes. **d** Integrative Genomics Viewer (IGV) screenshot presenting the g.2205625C>CG variant in the two whole-genome sequenced cases. **e** Predicted wild-type (wt) and IC cDNA e protein. **f** Schematic representation of the bovine FA2H protein and its two domains and the identified pathogenic frameshift variant (p.Ala4fsTer142; red arrow)

Pedigree records allowed the identification of a common ancestor as all IC-affected Chianina cattle were inbred from a sire born in 1976 (Online Resource 3). Pedigree analysis was consistent with monogenic autosomal recessive inheritance, and therefore carried out homozygosity mapping as all cases would likely be homozygous for a common chromosome segment flanking the causal mutation. This revealed a total of two identical-by-descent (IBD) segments shared by all seven cases with available SNP data (case 1–7): one 548 kb-sized region on chromosome 5 from 21.75 to 22.298 Mb and a second 1.92 Mb-sized region on chromosome 18 from 1.37 to 3.29 Mb (Fig. 3a).

Filtering of WGS for private shared homozygous variants present in sequenced genomes of cases 1 and 6 and absent in 597 available control genomes identified ten private protein-changing variants with a predicted moderate or high impact. Analyzing the occurrence of these variants in the global control cohort of 4110 genomes of a variety of breeds (Hayes and Daetwyler 2019), a single frameshift variant in *FA2H* with a predicted high impact on the encoded protein exclusively present in the genome of the case 1 and 6 remained. The homozygous variant in *FA2H* exon 1 on chromosome 18 (chr18:g.2205625C>CG; c.9dupC) was confirmed using IGV software (Fig. 3b, d). The deleterious *FA2H* variant (NM_001192455.1: c.9dupC) was predicted to result in a frameshift in the beginning of the protein after alanine 4 with a stop codon after aspartate 142 (NP_001179384.1: p.Ala4ArgfsTer142) resulting in a completely different amino acid sequence, if expressed, when compared with the wild type protein (Fig. 3e, f).

### FA2H genotyping

To confirm and evaluate the presence of the *FA2H* variant, the affected genomic region was amplified by PCR and Sanger sequenced (Fig. 3c) in a total of ten cases, and presumable dams and sires when available. Analyzing the sequencing data, we observed that all cases were homozygous for the variants, whereas the available parents were heterozygous as expected for obligate carriers (Table 1). Furthermore, genotyping of 113 Chianina sires representing the active breeding population revealed a carrier ratio of 15% whereas the variant was absent in a global cohort of more than 4700 cattle of various breeds (Table 1).

**Table 1:** Association of the 1 bp duplication (c.9dupC) variant in *FA2H* with ichthyosis congenita (IC) in Chianina cattle

	Genotype		
	wt/wt	wt/dup	dup/dup
IC-affected cattle	0	0	10
Obligate carriers^a^	0	10	0
Chianina top sires	96	17	0
Normal control cattle from various breeds	4707	0	0

^a^Parents of the affected animal

## Discussion

Here we describe the clinicopathological phenotype displayed by nine Italian Chianina cattle with IC and present the results of the genetic analysis that identified a recessively inherited frameshift mutation in *FA2H*, providing a novel candidate gene for skin disorders in both humans and animals. Furthermore, we provide a DNA-based diagnostic test that enables the selection against this sub-lethal allele that show an estimated frequency of ~ 7.5% in Chianina top sires.

Clinicopathological resemblances between familial forms of ichthyosis in humans and animals and the keratinization defect observed histologically in IC-affected Italian Chianina cattle led to the hypothesis that genetic variants in candidate genes for ichthyosis could be responsible for this disease in cattle. However, protein-changing variants within these more than 30 known candidate genes (Oji et al. 2010) were not found within the two mapped IBD regions, thereby excluding these as likely candidates. We then performed whole-genome sequencing of two IC-affected cattle that led to the identification of a frameshift mutation in *FA2H* (c.9dupC; p.Ala4ArgfsTer142). The genetic association of this variant with the bovine familial IC phenotype was confirmed by the homozygous genotype in eight additionally affected cattle, including an older case presented in 2006, and by its absence in all other sequenced genomes. Furthermore, reported expression of *FA2H* transcripts in the urinary tract supports the associated urolithiasis and cystitis seen in some of the IC-affected cattle. Finally, the predicted consequence of the frameshift variant demonstrating a loss-of-function supports causality. This variant is therefore the first in any domestic animal species to be associated with IC, and *FA2H* should be considered an additional candidate gene for syndromic forms of ichthyosis in humans.

While genetic analysis strongly suggested the association of p.Ala4ArgfsTer142 allele with IC in affected Italian Chianina cattle, the frameshift variant lies very near the N-terminal end of the protein. Therefore, the impact of such a significant truncation on protein function is probably high, thus the variant represents a most likely pathogenic loss-of-function mutation. Within a representative cohort of the current Italian Chianina population, a moderate allele frequency and the absence of the homozygous genotype for the deleterious allele was noticed.

In humans, mutations in *FA2H* (OMIM611026) are associated with recessively inherited spastic paraplegia type 35 (Dick et al. 2010), leukodystrophy with spasticity and dystonia (Edvardson et al. 2008), and fatty acid hydroxylase-associated neurodegeneration, a rare subtype neurodegeneration with brain iron accumulation (Kruer et al. 2010). So far, more than 40 different mutations have been associated with these neurological phenotypes (Rattay et al. 2019; Kawaguchi et al. 2020). However, to the best of our knowledge, no pathogenic variant in the *FA2H* associated to a form of ichthyosis has been reported in both animal and human species. Therefore, our study in cattle provides the first large-animal model of an *FA2H*-related congenital skin disorder.

*FA2H* encodes the endoplasmic reticulum enzyme fatty acid 2-hydroxylase, which plays a major role in the de novo synthesis of sphingolipids containing 2-hydroxy fatty acids (Alderson et al. 2004, 2005; Maldonado et al. 2008). 2-Hydroxy sphingolipids are very plentiful in neural tissue since the major components of myelin are galactolipids (galactosylceramide and sulfatide) with 2-hydroxy fatty acids (Alderson et al. 2005; Maldonado et al. 2008). However, the function of 2-hydroxyl modification of sphingolipids is still poorly known, although several studies evidently demonstrated that these compounds (including ceramides) play important roles in signal transduction (Hannun and Obeid 2008). Particularly, a study demonstrated that absence of FA2H lead to the impairment of cAMP-dependent cell cycle exit of Schwannoma cells, suggesting that FA2H sphingolipids may negatively regulate the cell cycle (Alderson and Hama 2009). Moreover, *FA2H* is highly expressed in the epidermis (Uchida et al. 2007). Notably, mammalian skin contains reasonably large amounts of 2-hydroxylated sphingolipids, which are involved in cell–cell recognition, signal transduction, and intercellular adhesion (Hakomori 2002; Uchida et al. 2007). The sphingolipids' ceramide backbone also plays a role as an intracellular signal of cell arrest, cellular senescence, and apoptosis in several types of cell, including keratinocytes (Hannun and Luberto 2000). Besides these ubiquitous bioregulatory functions, ceramide are abundant components of the extracellular lamellar membranes in the outermost layers of the epidermis, such as the stratum corneum, where they play an important role in the epidermal permeability barrier function (Holleran et al. 2006). Also, a notable increase in ceramide is noticed during epidermal differentiation (Holleran et al. 2006). In humans, it is known that differentiation-dependent up-regulation of ceramide synthesis and fatty acid elongation is accompanied by up-regulation of FA2H. Furthermore, the 2-hydroxylation of fatty acid by FA2H occurs prior to generation of ceramides/glucosylceramides, and 2-hydroxyceramides/2-hydroxyglucosylceramides are essential for epidermal lamellar membrane formation (Uchida et al. 2007). Such findings suggest that the expression of FA2H is essential for epidermal permeability barrier homeostasis and responsible for synthesis of 2-hydroxylated sphingolipids in keratinocytes of mammalian skin. Therefore, the severe orthokeratotic hyperkeratosis observed in the Chianina cattle with IC could be a consequence of the frameshift insertion in the *FA2H* gene. Notably, lack of FA2H in *Fa2h*^−/−^ mice leads to hyperproliferation of sebocytes and enlarged sebaceous glands during hair follicle morphogenesis and anagen (active growth phase) in adult mice (Maier et al. 2011). Interestingly, the IC-affected animals included in this study often displayed intracytoplasmic, spindle-shaped, optically empty clefts within the sebocytes, although the sebaceous glands were similar in size to the ones observed in control animals. Sebaceous glands are holocrine glands that secrete a viscous, lipid-rich fluid rich in cholesterol and wax esters, triglycerides, squalene and cholesterol playing an important role in thermoregulation (Porter 2001). The rate of sebum is associated with the number and size of glands, and low production of sebum might lead to sebatosis or xerosis (Porter 2001; Shamloul and Khachemoune 2020). The major functions of sebum are to lubricate the skin and hair conferring impermeability to water, and in thermoregulation (Shamloul and Khachemoune 2020). Moreover, sebaceous glands play a role in immunity since sebum is thought to have antibacterial and antifungal properties (Strauss et al. 1983). Consequently, our findings may suggest that cattle affected by IC might be predisposed to develop skin secondary infections and present thermoregulation deficits due to this genetic defect. These situations were evidently suspected for two of our patients. Moreover, in *Fa2h*^−/−^ mice, deficiency in *Fa2h* caused a delay in emergence of the fur during morphogenesis and depilation-induced anagen and a cyclic alopecia (Maier et al. 2011). Herein, the cases revealed a localized alopecia, but the hair follicles present in the biopsies taken from the IC cutaneous lesions were unremarkable.

In mice, depletion of FA2H decreases the protein levels of GLUT4 leading to reduced glucose uptake and lipogenesis under basal and insulin-stimulated conditions (Guo et al. 2010). GLUT4 deficiency in mice (Slc2a4^tm1Mch^/Slc2a4^tm1Mch^) leads to retarded growth, decreased expected longevity and abnormal cellular glucose and fat metabolism (Katz et al. 1995). Intriguingly, all Chianina cattle homozygous for the *FA2H* mutation showed retarded growth and decreased expected longevity. Unfortunately, metabolic analysis to access the glucose metabolism was not performed.

Beside the skin lesions that were displayed in all the cases, four out of ten cases showed urolithiasis and three out of ten revealed cystitis. It is worth to highlight that in the six animals where these findings were not recorded: one was euthanized 2 days after birth, and consequently, the absence of these findings might be explain by the young age of the calf; the remaining five were clinically examined only at the farms and, therefore, it was not possible to have a follow-up of the clinical status. On the contrary, the cases where we observed urolithiasis and cystitis were recovered at the clinic allowing the performance of a more detailed examination. Urolithiasis is a multifactorial disease resulting from complex interactions between environmental and genetic factors. Interestingly, *FA2H* in humans is also expressed in the urinary bladder and kidney (Fagerberg et al. 2014). However, physiological functions of FA2H in these organs are largely unknown.

## Conclusions

Rare disorders like IC in livestock are usually not reported or are mis-diagnosed. Based on the known function of *FA2H* and its role in the skin, the predicted impact of the identified variant and its perfect co-segregation with the disease phenotype in the studied pedigree, we conclude that inherited IC in Chianina cattle is caused by a homozygous loss-of-function variant in *FA2H*. Thereby, this study represents an outstanding animal model for the understanding of similar conditions in different species and adds *FA2H* to the list of candidate genes for ichthyosis in humans. This example highlights the utility of precision diagnostics including genomics, for understanding rare disorders and the value of surveillance of cattle breeding populations for harmful genetic disorders.

Moreover, this study provides a DNA-based diagnostic test that allows selection against the identified pathogenic variant in the Chianina cattle population. Due to the high economic value of many Chianina cattle, including their skin for leather production, genetic testing should be pursued to prevent breeding of carriers that produce affected calves.

## Supplementary Information

Below is the link to the electronic supplementary material.

Supplementary file1 (DOCX 13 KB)

Supplementary file2 (XLSX 14 KB)

Supplementary file3 (XLSX 28 KB)

Supplementary file4 (TIF 1265 KB)

## Data Availability

The whole-genome data of our group have been made freely available under study accession number PRJEB28191in the European Nucleotide Archive (http://www.ebi.ac.uk/ena). All accession numbers of the WGS are available in the Online Resource 2. SAMEA7690197 and SAMEA7690198 are the samples accession number of case 1 and case 6, respectively. All references to the bovine FA2H gene correspond to the NCBI accessions NC_037345.1 (chromosome 18, ARS-UCD1.2), NM_001192455.1 (*FA2H* gene), and NP_001179384.1 (FA2H protein). For the protein structure of FA2H, the UniProt database accession number E1BGC2 was used.
