# Supplementary material for: A frameshift insertion in FA2H causes a recessively inherited form of ichthyosis congenita in Chianina cattle
Source: Mol Genet Genomics. 2021 Oct 2;296(6):1313–22. doi: 10.1007/s00438-021-01824-8 (PMC8550120; doi:10.1007/s00438-021-01824-8)
Supplement: Supplementary file 1 — Supplementary file1 (DOCX 13 KB) [file 438_2021_1824_MOESM1_ESM.docx]

**Online Resource 1** (.xlsx) Phenotype records and genotypes of 10 Chianina cattle affected by ichthyosis congenita.

**Online Resource 2** (.xlsx) List of EBI Accession numbers of all publicly available genome sequences.

**Online Resource 3** (.tiff) Pedigree of the Chianina cattle affected by ichthyosis congenita.
