# Supplementary figures and images for: A frameshift insertion in FA2H causes a recessively inherited form of ichthyosis congenita in Chianina cattle
Source: Mol Genet Genomics. 2021 Oct 2;296(6):1313–22. doi: 10.1007/s00438-021-01824-8 (PMC8550120; doi:10.1007/s00438-021-01824-8)

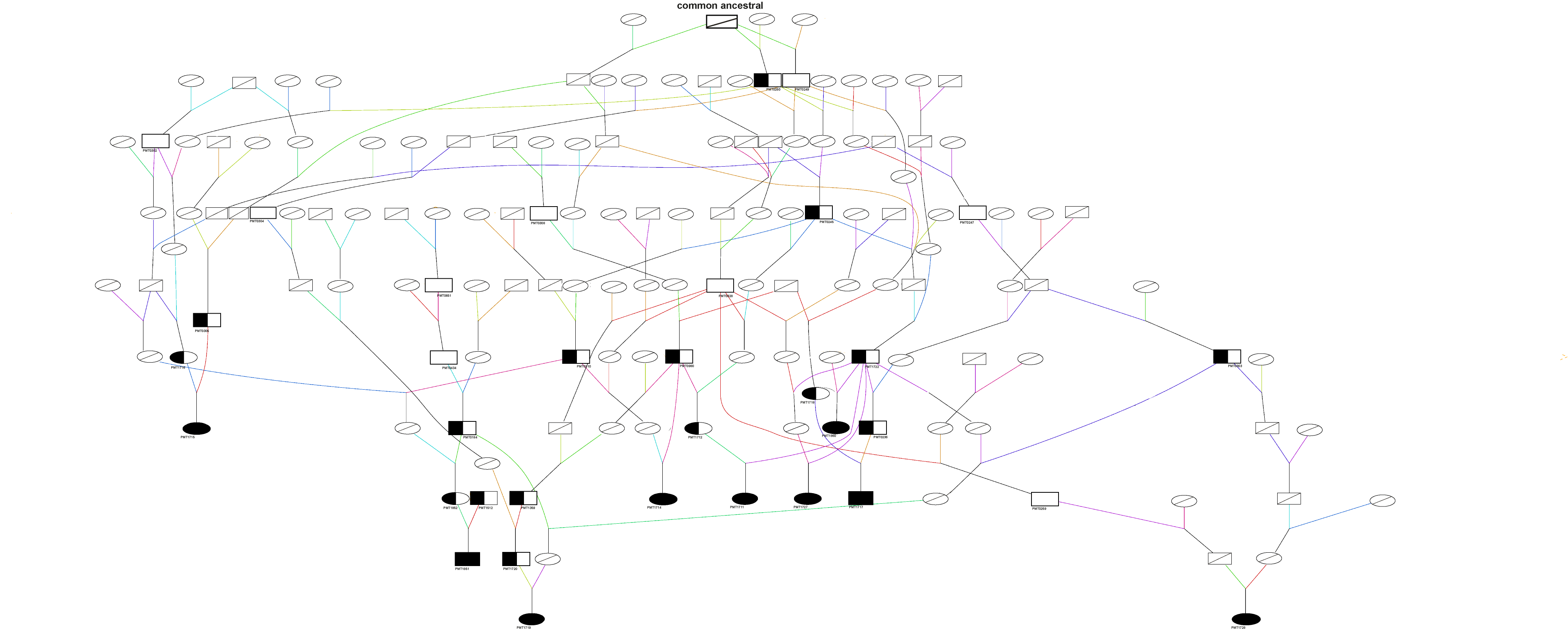

Supplement: Supplementary file 4 — Supplementary file4 (TIF 1265 KB) [file 438_2021_1824_MOESM4_ESM.tif]
